# Insights into the Inhibitory Mechanism of Viniferifuran on Xanthine Oxidase by Multiple Spectroscopic Techniques and Molecular Docking

**DOI:** 10.3390/molecules27227730

**Published:** 2022-11-10

**Authors:** Yaxin Yang, Qian Chen, Shiyang Ruan, Junli Ao, Shang-Gao Liao

**Affiliations:** 1 School of Basic Medicine, Guizhou Medical University, Guian New District, Guizhou 550025, China; 2 State Key Laboratory of Functions and Applications of Medicinal Plants & School of Pharmacy, Guizhou Medical University, Guian New Area, Guizhou 550025, China

**Keywords:** viniferifuran, xanthine oxidase, inhibition mechanism

## Abstract

Viniferifuran was investigated for its potential to inhibit the activity of xanthine oxidase (XO), a key enzyme catalyzing xanthine to uric acid. An enzyme kinetics analysis showed that viniferifuran possessed a strong inhibition on XO in a typical anti-competitive manner with an IC_50_ value of 12.32 μM (IC_50_ for the first-line clinical drug allopurinol: 29.72 μM). FT-IR and CD data analyses showed that viniferifuran could induce a conformational change of XO with a decrease in the *α*-helix and increases in the *β*-sheet, *β*-turn, and random coil structures. A molecular docking analysis revealed that viniferifuran bound to the amino acid residues located within the activity cavity of XO by a strong hydrophobic interaction (for Ser1214, Val1011, Phe914, Phe1009, Leu1014, and Phe649) and hydrogen bonding (for Asn768, Ser876, and Tyr735). These findings suggested that viniferifuran might be a promising XO inhibitor with a favorable mechanism of action.

## 1. Introduction

Hyperuricemia, a disease resulting from uric acid overproduction or underexcretion, has been a major threat to human health [1,2,3]. Xanthine oxidase (XO) is an enzyme responsible for the catalytic oxidation of xanthine to uric acid [4,5,6]. The inhibition of XO has been proven to be one of the most effective strategies to diminish uric acid production for the treatment of hyperuricemia and other XO-related diseases [7]. Several XO inhibitors such as allopurinol and febuxostat are existing first-line drugs used to treat hyperuricemia and gout [8,9], yet their side effects (including renal toxicity, hypersensitivity syndrome, vasculitis, and Stevens–Johnson syndrome) greatly limit their long-time application [10,11]. Thus, the discovery of new XO inhibitors with a more acceptable safety profile is of great importance for the treatment of hyperuricemia.

Chinese herbal medicines (CHMs) are an important source for drug discoveries. A number of bioactive compounds from CHMs—including terpenoids, flavonoids, phenylethanoids, and alkaloids—have been demonstrated to be promising XO inhibitors [12]. Understanding the inhibitory behavior and mechanism of action of these XO inhibitors is of benefit for the development of XO inhibitors as anti-hyperuricemia and anti-gout agents.

Fourier-transform infrared spectroscopy (FT-IR) and circular dichroism (CD) spectroscopy are currently two key spectroscopic techniques for the characterization of interactions between proteins and their ligands [13]. In addition, molecular docking has also been adopted as a typical technique to evaluate the binding mechanism of proteins and their ligands [13].

**Figure 1:**
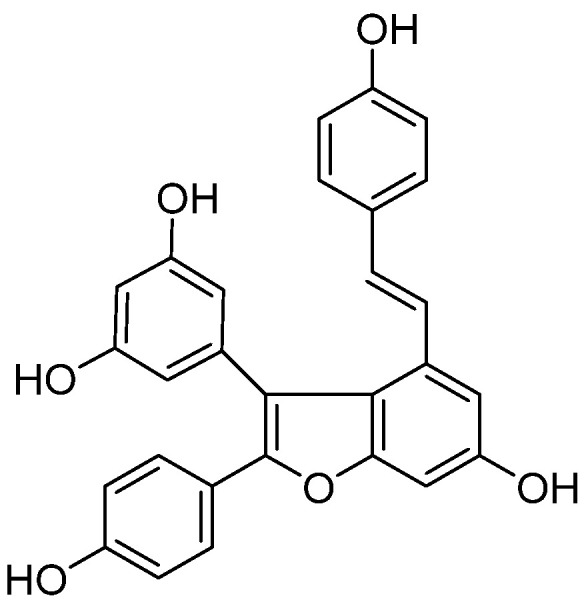
Chemical structure of viniferifuran.

Viniferifuran (Figure 1) is a natural product that was isolated from the roots of *Caragana sinica* as a potent XO inhibitor in our previous work [14,15]. However, its interaction with the target protein XO was unknown. In the current investigation, the inhibitory mechanism and the inhibitory mode of viniferifuran on XO were studied by a combination of an enzyme kinetic analysis, spectroscopic (FT-IR and CD) analyses, and molecular docking.

## 2. Results and Discussion

### 2.1. XO Inhibitory Activity of Viniferifuran

**Figure 2:**
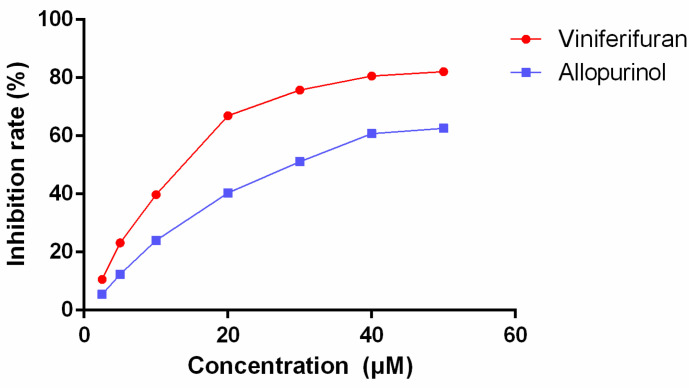
Inhibitory effect of viniferifuran and allopurinol on XO.

The inhibitory rates of different concentrations of viniferifuran and allopurinol (Figure 2) showed that both compounds possessed significant XO inhibitory effects (IC_50_ values of 12.32 and 29.72 μM, respectively, for viniferifuran and allopurinol). The results demonstrated that viniferifuran was a more potent XO inhibitor compared with the clinical drug allopurinol.

### 2.2. Inhibition Mechanism of Viniferifuran on XO

**Figure 3:**
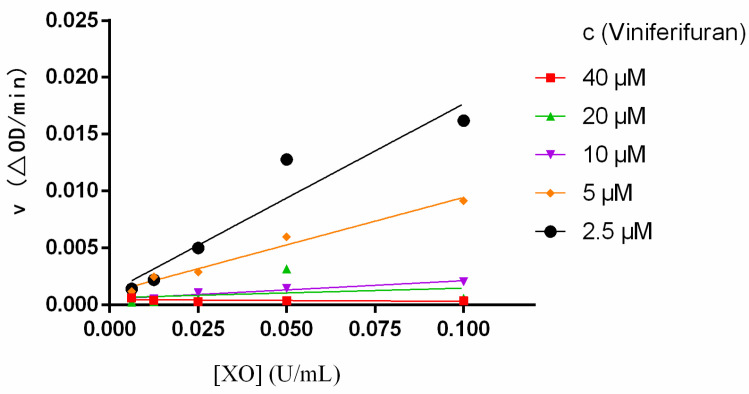
Plots of *ν* vs. XO. Each point is the mean of three independent determinations.

The reversibility of the inhibition of viniferifuran on XO was evaluated by building *ν* vs. XO plots at diverse viniferifuran concentrations [13]. As depicted in Figure 3, the plots displayed an acceptable linearity at all concentrations through the origin point. Additionally, the slope of the line decreased as the concentration of viniferifuran increased, which indicated that the presence of viniferifuran did not decrease the amounts of XO, but rather caused an overall reduction in its activity with respect to the xanthine oxidation. These findings demonstrated that the XO inhibition of viniferifuran was reversible [16].

### 2.3. Inhibition Mode of Viniferifuran on XO

**Figure 4:**
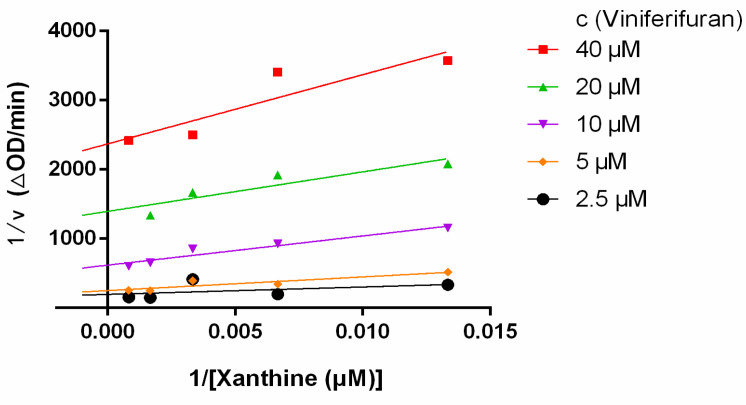
Lineweaver−Burk plots.

**Figure 5:**
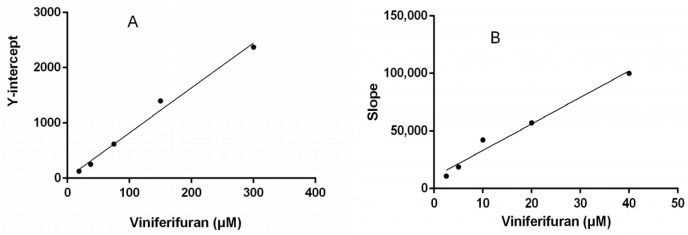
(**A**) Secondary plots of the Y-intercept of the Lineweaver–Burk plots vs. viniferifuran. (**B**) Secondary plots of the slopes of the Lineweaver–Burk plots vs. viniferifuran. Each point is the mean of three independent determinations.

Lineweaver–Burk plots were constructed to assess the inhibitory kinetics of viniferifuran on XO. The results (Figure 4) indicated that each line was nearly parallel, suggesting that viniferifuran inhibited XO in an anti-competitive inhibition manner [8,17]. In addition, a good linear fitting of their secondary plots (Y-intercept vs. viniferifuran and slope vs. viniferifuran) (Figure 5) demonstrated that viniferifuran had one specific binding site on XO [18].

### 2.4. Secondary Structure Analysis by FT-IR

**Figure 6:**
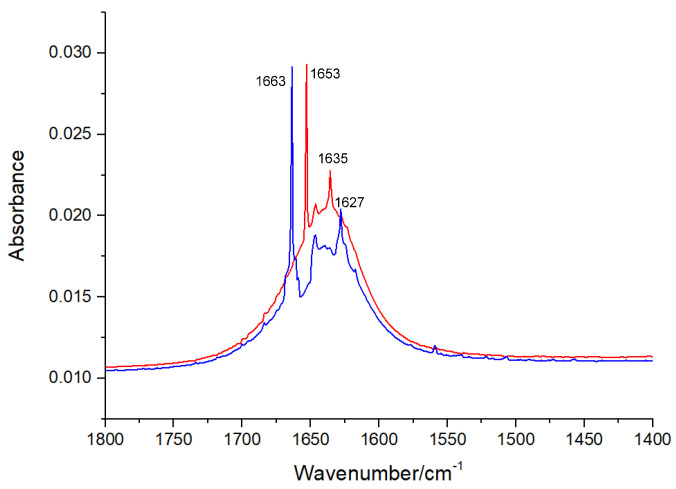
Extracted FT-IR spectra (1800 to 1400 cm^−1^) of XO (red) and viniferifuran–XO solution (blue) at pH 7.0 and room temperature. The concentrations of XO and viniferifuran were 0.1 U/mL and 10 μM, respectively.

**Figure 7:**
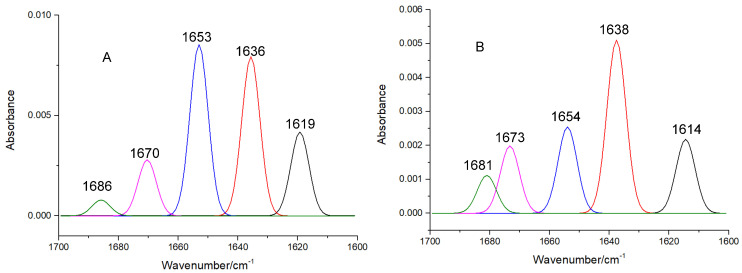
The curve-fitted amide band I of free XO (**A**) and viniferifuran–XO (**B**).

An FT-IR spectral analysis is a useful method for the determination of the secondary structures of proteins. In the FT-IR spectra of proteins, typical amide bands associated with different vibrations of the peptide moiety can be observed. During the study of the secondary structures of proteins, amide bands I (1700–1600 cm^−1^, originating from the C=O stretching vibration) and II (1600–1500 cm^−1^, caused mainly due to C—N stretching and N—H in-plane bending) are the principal vibrational bands of a peptide moiety [19]. As depicted in Figure 6 and Figure 7, after the treatment with viniferifuran, the amide band I of XO moved from 1653 to 1663 cm^−1^ and its amide band II shifted from 1635 to 1627 cm^−1^. A similar variation was reported for the FT-IR spectrum of genistein-mediated XO [20].

The high sensitivity of amide band I makes it an ideal signal for investigating the secondary structures of proteins. IR absorptions in the regions of 1615–1637 cm^−1^ (for *β*-sheet), 1638–1648 cm^−1^ (for random coil), 1649–1660 cm^−1^ (for *α*-helix), 1661–1680 cm^−1^ (for *β*-turn), and 1681–1692 cm^−1^ (for *β*-antiparallel) have been successfully used to assign contents to their corresponding secondary structures [21]. As shown in Table 1, the formation of the viniferifuran–XO complex increased the contents of *β*-sheet, *β*-turn, *β*-antiparallel, and random coil from 38.73%, 18.06%, 1.60%, and 15.43% to 41.08%, 27.71%, 3.18%, and 15.72%, respectively, and decreased the content of *α*-helix from 19.31% to 6.59%.

### 2.5. Secondary Structure Content Analysis by CD

**Figure 8:**
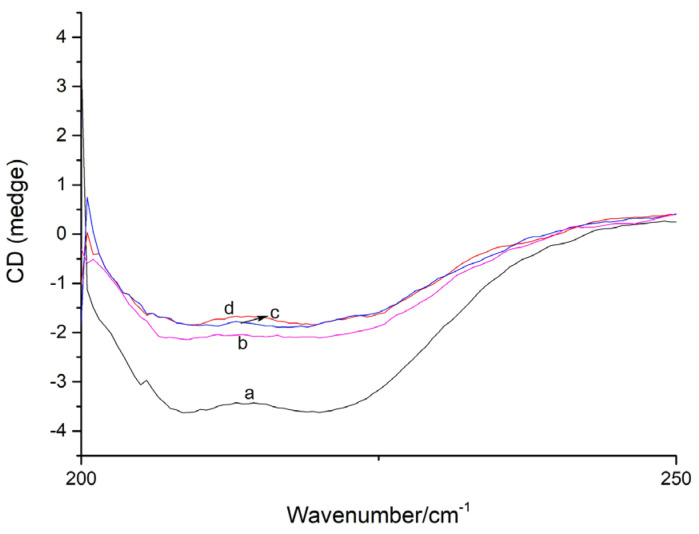
The CD spectra of XO and viniferifuran–XO. XO = 0.1 U/mL; viniferifuran = 0, 1, 5, and 10 μM for curves a to d, respectively.

A CD analysis has become an effective method to identify the secondary structure of proteins. As indicated in the far-UV CD spectra (Figure 8), two negative peaks at 210–230 nm, characteristic of the peptide bonds of *α*-helix and *β*-sheet, were observed for free XO [22,23,24]. The formation of the viniferifuran–XO complex obviously reduced the intensity, but had no significant effects on the peak shape and position, suggesting a conformational alteration of XO after the viniferifuran treatment.

Additionally, the content of the secondary structure of XO was calculated using the online SELCON3 platform. As shown in Table 2, the addition of viniferifuran to form a viniferifuran–XO complex led to a significant decrease in *α*-helix, marked increases in *β*-sheet and *β*-turn, and a slight increase in random coil. The tendency was strengthened by increasing the concentration of viniferifuran, where the content of *α*-helix decreased from 12.83 % to 5.25%; those of *β*-sheet, *β*-turn, and random coil increased from 41.44%, 17.73%, and 18.36% to 44.08, 29.63, and 18.75%, respectively, when the molar ratios of viniferifuran to XO increased from 1:1 to 10:1. These results indicated that viniferifuran could induce conformational changes in XO from an *α*-helix-rich secondary structure toward a *β*-helix-relatively rich one.

### 2.6. Binding Mode Analysis by Molecular Docking

**Figure 9:**
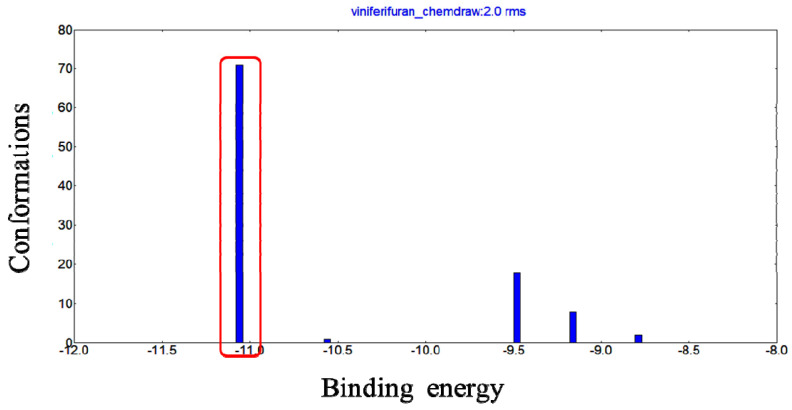
Conformational clusters of viniferifuran–XO (PDB ID: 1N5X) from 100 docking runs.

Molecular docking has been frequently employed to analyze the binding mode of ligands and proteins. The docking results (Figure 9) showed that when the RMSD tolerance was set at 2.0 Å, 100 docking runs could be grouped as 5 conformational clusters. The cluster with the most minimal energy and most frequent locus was selected for the binding analysis. The docking results showed that the most minimal binding energy was −11.06 kcal mol^−1^.

**Figure 10:**
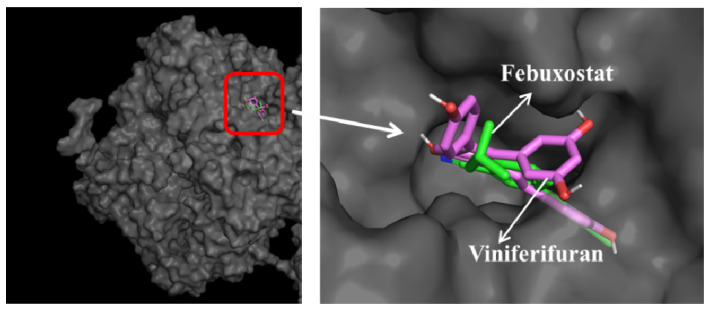
Docking results of viniferifuran (pink-purple)–XO and febuxostat (green)–XO and superimposed docking conformations of viniferifuran and febuxostat in the active pocket of XO.

The Mo-pt domain is the functional site of XO in which the oxidation of xanthine to uric acid occurs; Arg880, Phe1009, Phe914, Glu802, Asn768, Thr1010, Val1011, Leu873, and Glu1216 are its critical amino acids [25]. The docking results showed that viniferifuran could enter the active pocket to bind to the Mo-pt domain (Figure 10). When binding to the active pocket, viniferifuran was shown to be adjacent to a few hydrophobic residues (Ser1214, Val1011, Phe914, Phe1009, Leu1014, and Phe649), suggesting that hydrophobic interactions might play a critical role in the inhibitory behavior of viniferifuran against XO. In addition, the observation of three hydrogen bonds between the free hydroxyl groups of viniferifuran and the residues Asn768, Ser876, and Tyr735 of XO suggested that hydrogen bonding might also be involved in their efficient interaction.

**Figure 11:**
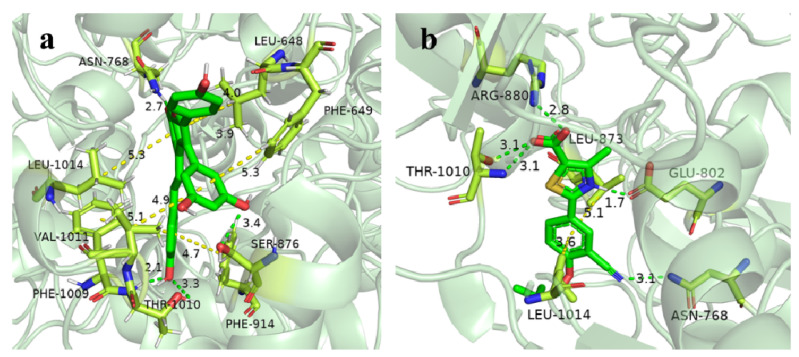
Close-up view of the binding mode of viniferifuran in the active site of XO (**a**) and febuxostat in the active site of XO (**b**).

A comparison of the docking results of febuxostat and viniferifuran (Figure 11 and Table 3) showed that viniferifuran had a relatively lower binding energy (−11.6 kcal mol^−1^ vs. −10.8 kcal mol^−1^) and that both ligands shared one hydrogen-forming amino acid residue (Asn768) with a close interaction distance (2.7 Å for viniferifuran vs. 3.3 Å for febuxostat) in the active site of the target protein. Several critical amino acids (Phe1009, Phe914, Asn768, and Val1011 for the viniferifuran–XO complex; Arg880, Glu802, Asn768, Thr1010, and Leu873 for the febuxostat–XO complex) of the Mo-pt domain were all involved in the interactions of both complexes. These observations suggested that viniferifuran and febuxostat shared the same binding site and had similar interactions with their target protein XO. However, the viniferifuran–XO complex showed more hydrophobic interactions and a much shorter hydrophobic interaction distance (as low as 3.7 Å compared with 4.8 Å for febuxostat–XO). Although one less hydrogen bond was observed for the viniferifuran–XO complex, the close hydrogen bond distance (2.7 Å with Asn768 in the viniferifuran–XO complex and 2.8 Å with Arg880 for the febuxostat–XO complex) observed for the two complexes suggested that both ligands had close hydrogen bonding interactions. These observations suggested that both stronger hydrophobic and hydrogen bonding interactions between viniferifuran and XO were responsible for the potent inhibitory effect of viniferifuran on XO.

## 3. Materials and Methods

### 3.1. Chemicals and Reagents

XO (CN 379122, 100 U/mg), xanthine, and allopurinol were acquired from J&K Scientific Ltd. (Beijing, China). The stock solutions of XO and xanthine were prepared with phosphate buffered saline (pH 7.4). The stock solution of viniferifuran was prepared by dissolving viniferifuran in dimethyl sulfoxide (DMSO) (analytical grade) and was diluted with a PBS buffer when needed. The concentrations of DMSO were below 4% in the whole experiment [23]. All reagents and solvents were of an analytical grade and ultrapure water was obtained through a Milli-Q water purification system (Millipore, Burlington, MA, USA).

**Figure 12:**
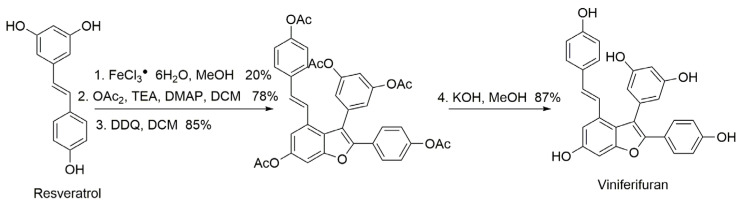
The synthesis route of viniferifuran.

Viniferifuran was synthesized according to the method of Elofsson and coworkers [24,26]. The synthetic route is shown in Figure 12, where resveratrol was used as the starting material to obtain the target compound viniferifuran with an overall yield of about 11%.

### 3.2. XO Activity Assay

The XO inhibition assay was conducted in 96-well plates according to our previous method [23]. Briefly, to the reaction medium (200 μL) in each well of the 96-well plates, 50 μL of XO (0.025 U/mL^−1^) and 100 μL of different concentrations of viniferifuran (dissolved in 4% DMSO-PBS) were added. The mixture was preincubated for 20 min at 25 °C. Subsequently, 50 μL of xanthine (150 μM) was added to initiate the reaction system at 37 ℃. Uric acid formation was monitored at 290 nm at 25 min using a Shimadzu UV-2450 spectrophotometer (Japan). Allopurinol and PBS served as the positive control and blank control, respectively. The inhibition rates of the XO inhibitors were calculated by dividing the slope of the reaction kinetics equation obtained by the reaction with the inhibitor by that obtained without the inhibitor [27,28,29].

The concentration of inhibitor required for a 50% inhibition (IC_50_) of XO was used to evaluate the inhibition extent of the inhibitor and was calculated by GraphPad Prism 6 (GraphPad Software, San Diego, CA, USA).

### 3.3. Measurement of Inhibition Mechanism and Inhibition Mode

The inhibitory mode of viniferifuran against XO was determined from the Lineweaver–Burk plot of samples with xanthine, XO, and viniferifuran (at different concentrations) [10]. The xanthine concentration was maintained at 150 μM; that of XO ranged from 0.00625 to 0.1 U/mL^−1^. The enzyme activity was determined for all samples and the enzyme activity and concentration mapping were used to deduce the inhibition mechanism. The inhibition mode of viniferifuran was evaluated by maintaining XO at 0.025 U/mL with varying concentrations of xanthine (75, 150, 300, 600, and 1200 μM). The enzyme activity was determined at different concentrations of viniferifuran (2.5, 5, 10, 20, and 40 μM). The inhibition mode was described by the Lineweaver–Burk equation in a double-reciprocal form [30,31,32]. GraphPad Prism 6.0 (USA) was used for the calculation.

### 3.4. FT-IR Experiments

The FT-IR experiments were carried out on an IR Tracer FT-IR spectrometer (Japan). All spectra in the region of 1800–1400 cm^−1^ were recorded with a resolution of 6 cm^−1^ and 60 scans in a PBS buffer (pH 7.4) at 25 ℃. XO solutions with or without viniferifuran were uniformly coated on KBr chips. The free viniferifuran (10 μM) and viniferifuran–XO (0.1 U/mL) solution spectra were corrected by subtracting the background spectra. PeakFit v4.04 was used to process the spectra through baseline subtraction, smoothing, second derivative spectrum deconvolution, and curve-fitting to analyze the contents of the different secondary structures of XO [13].

### 3.5. Circular Dichroism Analysis

Through a 1.0 mm quartz cuvette and under a constant nitrogen flush, the CD spectra (200 to 250 nm) of XO were measured using a Bio-Logic MOS 450 CD spectrometer (France). The concentration of XO was maintained at 0.1 U/mL and the concentrations of viniferifuran were set as 0, 1, 5, and 10 times that of XO. All CD spectra were subtracted for the baseline corrections. Additionally, the contents of the different secondary structures of the enzyme were calculated using the online SELCON3 program (http://dichroweb.cryst.bbk.ac.uk/html/home.shtml, accessed on 1 November 2022) [13].

### 3.6. Molecular Docking Studies

The rationale for the use of viniferifuran as a potent XO inhibitor and its binding mode in the active sites of XO was further investigated and demonstrated by molecular docking. From the Protein Data Bank (http://www.rcsb.org/pdb, accessed on 1 November 2022), the X-ray crystal structure of the XO–febuxostat complex (PDB ID: 1N5X) was downloaded [12,33]. As 1N5X contains two protein chains (chains A and B) and each chain has an identical protein sequence, only chain A was used for docking. Protein (1N5X) protonation was carried out by an online service (https://server.poissonboltzmann.org/pdb2pqr, accessed on 1 November 2022) and the water molecules and ligand (febuxostat) were removed; the missing hydrogen atoms, Gasteiger charges, and non-polar hydrogens were merged into the carbons. All atoms were then assigned an AD4 type for the docking preparation. The 3D structure of viniferifuran was produced using Chem3D Ultra 14.0. The molecular docking was performed using AutoDock 4.2. Default settings, except genetic algorithm runs (100) and the Lamarkian genetic algorithm (with a local search), were used for the docking. During the docking process, a grid box of 60 Å × 60 Å × 60 Å and a grid spacing of 0.375 Å were defined. The docking model with the highest score was deemed to be the most favorable binding mode. The docking results were further visualized with PyMOL to show the interactions between viniferifuran and XO [7].

## 4. Discussion and Conclusions

The current investigation used enzyme kinetics, spectroscopic analyses, and molecular docking techniques to figure out the mechanism of how viniferifuran acted as an XO inhibitor. The findings indicated that viniferifuran had a substantial inhibitory effect, with an IC_50_ lower than the first-line clinical drug allopurinol. The inhibition of the catalytic activity of XO by viniferifuran proved to be reversible. Molecular docking and inhibition mode studies suggested that by binding to a single binding site of the activity cavity, viniferifuran elicited its XO inhibitory effect in a typical anti-competitive manner [34]. The potent XO inhibition of viniferifuran may be attributed to the desired and effective conformational change of the active center induced by its preferential active site binding, as indicated in the FT-IR and CD assays, in which the original *α*-helix-rich secondary structure of XO was changed toward a *β*-helix-relatively rich one upon the addition of viniferifuran. Increases in *β*-sheet, *β*-turn, and random coil indicated that the binding of viniferifuran to XO could break the hydrogen bonds in the protein chain to induce the distortion of the helix structure of *α*-helix, resulting in alteration to the secondary structure of the of the protein for effective binding [29]. In addition, a decrease in the content of *α*-helix could lead to the loss of its stability, eventually causing a decrease in the catalytic activity of XO [28,35].

Simultaneously, a molecular docking analysis revealed that viniferifuran had the same binding region as the first-line clinical drug febuxostat. Three benzene rings of viniferifuran were embraced in the hydrophobic cavity with residues Ser1214, Val1011, Phe914, Phe1009, Leu1014, and Phe648; the resulting hydrophobic interactions between viniferifuran and XO could facilitate the stabilization of the cavity and the viniferifuran–XO complex. Hydrogen bonds were found to be present between the core structure of benzofuran and the residues Asn768, Ser876, and Tyr735, which indicated that hydrogen bonding was another stabilizing factor for the activity cavity and the viniferifuran–XO complex. The results also suggested that the benzofuran motif and the phenyl groups were all responsible for the potent XO inhibitory activity of viniferifuran. Stronger hydrophobic interactions and a relatively weaker hydrogen bond interaction between viniferifuran and XO (when compared with those of febuxostat–XO) suggested that viniferifuran and febuxostat were bestowed with different structural motifs for their potent XO inhibitory effect. As *α*-helix is stabilized by hydrogen bonding [13,19], viniferifuran and febuxostat seemed to inhibit the catalytic activity of XO through different conformational constraints.

In conclusion, with a tendency to form hydrogen bonds and a hydrophobic interaction with the amino acid residues, viniferifuran may easily bind to a specific binding site of XO in the activity cavity to form a stable viniferifuran–XO complex and cause conformational changes in the secondary structure of XO for an effective oxidation catalysis. Moreover, the occupation of viniferifuran in the active site of XO could probably impede the entrance of xanthine to the catalytic center, efficiently blocking the catalytic oxidation of xanthine to uric acid.

The findings indicated that viniferifuran is a promising XO inhibitor, with a desired mechanism of action. Studies toward the evaluation of its effectiveness as an anti-hyperuricemia agent are pending.

## Figures and Tables

**Table 1:** The contents of the secondary structures of free XO and viniferifuran–XO determined by FT-IR.

Viniferifuran (μM)	*α*-Helix (%)	*β*-Sheet (%)	*β*-Turn (%)	Random Coil (%)	*β*-Antiparallel (%)
0	19.31	38.73	18.06	15.43	1.60
10	6.59	41.08	27.71	15.72	3.18

**Table 2:** The contents of different secondary structures of XO and its viniferifuran complex calculated from CD data.

Molar Ratios of Viniferifuran:XO	*α*-Helix (%)	*β*-Sheet (%)	*β*-Turn (%)	Random Coil (%)
0:1	16.50	40.52	12.90	18.10
1:1	12.83	41.44	17.73	18.36
5:1	8.37	42.67	23.26	18.52
10:1	5.25	44.08	29.63	18.75

**Table 3:** The docking results of febuxostat and viniferifuran with 1N5X.

Ligands	Binding Energy	Hydrophobic Interaction	Hydrogen Bonds
Viniferifuran	−11.06 kcal mol^−1^	Ser1214 (3.7 Å), Val1011 (4.9 Å), Phe914 (4.7 Å), Phe1009 (5.1 Å), Leu1014 (5.3 Å), and Phe649 (5.3 Å)	Asn768 (2.7 Å), Ser876 (3.4 Å), and Tyr735 (5.5 Å)
Febuxostat	−10.18 kcal mol^−1^	Leu873 (5.1 Å) and Leu648 (4.8 Å)	Glu802 (5.1 Å), Thr1010 (3.1 Å), Arg880 (2.8 Å), and Asn768 (3.3 Å)
